# An Injectable Hyaluronic Acid-Based Composite Hydrogel by DA Click Chemistry With pH Sensitive Nanoparticle for Biomedical Application

**DOI:** 10.3389/fchem.2019.00477

**Published:** 2019-07-03

**Authors:** Xiaohong Hu, Ziyu Gao, Huaping Tan, Huiming Wang, Xincheng Mao, Juan Pang

**Affiliations:** ^1^School of Material Engineering, Jinling Institute of Technology, Nanjing, China; ^2^Biomaterials for Organogenesis Laboratory, School of Materials Science and Engineering, Nanjing University of Science and Technology, Nanjing, China

**Keywords:** hydrogel, Diels-alder click chemistry, pH-sensitive nanoparticle, cell scaffold, growth factor delivery

## Abstract

Hydrogels with multifunctional properties attracted intensively attention in the field of tissue engineering because of their excellent performance. Also, object-oriented design had been supposed to an effective and efficient method for material design as cell scaffold in the field of tissue engineering. Therefore, a scaffold-oriented injectable composite hydrogel was constructed by two components. One was pH-sensitive bifunctional nanoparticles for growth factor delivery to improve biofunctionability of hydrogel scaffold. The other was Diels-alder click crosslinked hyaluronic acid hydrogel as matrix. pH dependent release behavior of nanoparticle component was confirmed by results. And, its bioactivity was verified by *in vitro* cell culture evaluation. In consideration of high-efficiency and effectiveness, low toxicity, controllability and reversibility, dynamic covalent and reversible Diels-alder click chemistry was used to design a HA hydrogel with two kinds of crosslinking points. The properties of hydrogel like gelation time and swelling ratio were influenced by pH value and polymer concentration. Composite hydrogel was formed by *in situ* polymerization, which exhibited acceptable mechanical property as a scaffold for biomedical field. Lastly, *in vitro* evaluation from results of viability, DNA content and cell morphology confirmed that hydrogels could maintain cell activity and support cell growth. Compared with pure hydrogel, composite hydrogel possessed better properties.

## Introduction

Hydrogel, a water-swollen polymer network, is widely used in fields of biomedical fields due to their physiological-like aqueous environment (Bai et al., 2017; Celie et al., 2019; Massaro et al., 2019; Song et al., 2019; Zhao et al., 2019). Among its applications, scaffold-oriented hydrogel for biomedical application has an extra request of biofunctionability besides general characteristic of low toxicty and aqueous environment (Fu et al., 2012; Yu et al., 2013; Fan et al., 2015; Oh et al., 2016; Williams et al., 2017; Thanusha et al., 2018; Wang C. Z. et al., 2018; Zhu et al., 2018; Celie et al., 2019; Massaro et al., 2019; Song et al., 2019; Zhao et al., 2019). In consideration of natural component of extracellular matrix (ECM), hyaluronic acid (HA), as one of main component of ECM, is an optimal material to fabricate hydrogel for tissue engineering (Yu et al., 2013; Williams et al., 2017; Wang C. Z. et al., 2018; Zhu et al., 2018; Massaro et al., 2019). However, HA itself cannot form hydrogel naturally in solution due to absence of reactive groups or physical interactions. In early years, chemical crosslinkers were used to form HA hydrogels. But the toxicity of chemical crosslinker cannot satisfy the request of biocompatibility for cell scaffold, especially for that *in situ* formation scaffold. Recently, many efforts had been made to introduce function groups to HA main chain, which can be further crosslinked by their reaction though click chemistry, Michael addition and shift base reaction etc. (Yu et al., 2013; Bai et al., 2017). Although these attempts had been made great progress for HA hydrogel as cell scaffold, they could not be satisfied all requests as scaffold for all kinds of cells. Therefore, HA hydrogels with specific functions for specific cells and environment are also needed. Recently, as for crosslinking reaction, diels-alder clickchemistry has showed favorite characteristics for cell scaffold fabrication due to its low toxicity, high efficiency, moderate reaction condition, and reversible characteristic (Franc et al., 2009; Yu et al., 2013; Bai et al., 2017; Banerjee et al., 2017). Herein, HA hydrogel with self-healing property was designed through Diels-alder click chemistry as a cell scaffold to support cell growth.

Besides scaffold matrix properties, bioactive factors play important roles on cell proliferation and function expression (Akuta et al., 2015; Azevedo et al., 2015; Psarra et al., 2015; Muraoka et al., 2018). However, bioactive protein is liable to loss its function due to the transformation of secondary structure, which was induced by external stimuli. Therefore, one objective of our research is to realize the effective and efficient delivery of growth factor into cells, simultaneously maintaining the bioactivity of growth factor. Since heparin is a verified negative polysaccharide to protect the bioactivity of growth factor, it has been used in a great number of scaffold or carriers for growth factor protection (Psarra et al., 2015; Wu et al., 2015; Song et al., 2018). Another challenge is how to realize the precise delivery to cells. Stimulus from difference between intracellular and extracellular environment including pH value, enzyme, biotin-avidin interaction have been applied to carrier design and preparation for growth factor delivery. In view of mild acid condition of intracellular environment (pH 5.5–6.0), low pH responsive nanoparticle would be considered to be an effective carrier for growth factor delivery. Herein, a dual-structural pH sensitive nanocarrier for growth factor delivery was designed using pH-sensitive acetalated β-cyclodextrin (Ac-β-CD) as main body for pH response property and heparin nanogel as interpenetrating component for growth factor protection. The bioactivity of released growth factor was evaluated by *in vitro* cell behavior indicator like cell viability, DNA content and cell morphology. In the further step, the nanocarrier was incorporated with HA hydrogel to form composite hydrogel as cell scaffold, which was *in vitro* evaluated by 1-week cell growth.

## Experiment

### Material

4-(4,6-dimethoxy triazine)-4-methyl morpholine hydrochloride (DMTMM), furylmethylamine, adipicdihydrazide (ADH), maleimide modified PEG (mal-PEG-mal), 2-morpholinoethane sulfonic acid (MES), heparin (HEP), and 3-aminopropyl methacrylate (AMA) were purchased from Aladdin. Dimethyl sulfoxide (DMSO), dichloromethane (DCM), sodium periodate, ammonium persulfate (APS), N,N,N',N'-tetramethylethylenediamine (TEMED), and gelatin were purchased from Shanghai Chemical Industries Co. Ltd (China). Hyaluronate acid (HA, Mw = 1,000 kDa) was obtained from Shandong Furuida Co., China. Trypsin, Dulbecco's modified Eagle's medium (DMEM) and 3-(4, 5-dimethyl) thiazol-2,5-dimethyl tetrazolium bromide (MTT) were obtained from Sigma. Fetal bovine serum (FBS) was purchased from Sijiqing biotech. Co., China. PicoGreen dsDNA Assay Kit was bought from Thermo Fisher Scientific. Ac-β-CD was synthesized previously. All other reagents and solvents were of analytical grade and used as received.

### Synthesis and Characterization of Polysaccharide Derivatives

**Scheme 1:**
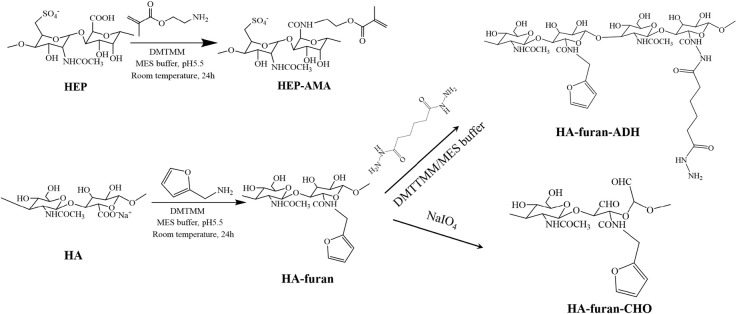
Scheme of synthesis for polysaccharide derivatives.

Synthesized processes of polysaccharide derivatives were shown in Scheme 1. HEP-AMA and HA-furan were synthesized by amidation between polysaccharides and functional chemical containing amine groups (furylmethylamine or AMA). Briefly, about 0.5 g HA or HEP was first dissolved in 150 mL 100 mM MES buffer solution. Seven hundred milligram of DMTMM were successively added into the solution to activate carboxyl groups for 30 min under magnetic stirring. After activation, 1.5 mmol functional chemical (furylmethylamine or AMA) was dropped or added to reaction system, which was continued to react for 24 h under dark at room temperature. Then the final solution was dialyzed with dialysis bag of 10 kDa cut-off molecular weight for 3 days to remove unreacted chemicals and byproduct of small molecule weight. Finally, polysaccharide derivative (HEP-AMA or HA-furan) was obtained by freeze drying at −60°C at a pressure of 7–8 Pa.

HA-furan-ADH was also obtained by amidation. Briefly, after 700 mg of DMTMM were used to activate carboxyl groups of 150 mL 3.3 mg/mL HA-furan buffer solution containing 100 mM MES for 30 min under magnetic stirring, 1.5 mmol ADH was added to react with HA-furan at room temperature for 24 h. Then HA-furan-ADH was finally obtained after the solution was dialyzed and freeze-dried according to above-mentioned conditions.

HA-furan-CHO was synthesized by oxidation of sodium periodate. Briefly, 5 mL 0.5 mol/L sodium periodate was dropped into 100 mL 5 mg/mL HA-furan solution to oxide HA. After the reaction continued for 2 h, 1 mL ethylene glycol was added to end the reaction. Then HA-furan-CHO was finally obtained after the solution was dialyzed and freeze-dried according to above-mentioned conditions.

These derivatives were characterized by ^1^H nuclear magnetic resonance (^1^H NMR, Bruker, AV500) using D_2_O as solvent. Grafting ratio of functional molecules for polysaccharides derivatives was calculated by ^1^H NMR spectra except grafting ratio of aldehyde group, which was qualified by the t-butyl carbazate assay.

### Preparation and Characterization of pH-Sensitive Bifunctional Nanoparticle

**Scheme 2:**
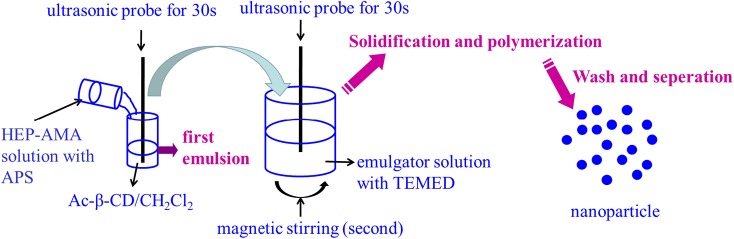
Scheme illustration to show the formation of nanoparticle.

pH-sensitive bifunctional nanoparticles were also fabricated by double emulsion method using previous synthesized pH-sensitive Ac-β-CD as a main material (Chen et al., 2017, 2018; Wang X. et al., 2018). The process of preparation was shown in Scheme 2. Briefly, 200 μL 40% HEP-AMA solution containing 50 mM APS was first emulsified into 1 mL of 10% w/v Ac-β-CD/DCM solution, which was further emulsified into 5 mL of 3% w/v gelatin aqueous solution containing 50 mM TEMED. Either first or second emulsion was formed under the help of sonication dispersion method, which was carried out by ultrasonic probe with 1 kW for 30 s per step. The obtained emulsion was immediately added into 20 mL of 1% w/v gelatin solution to evaporate DCM under magnetic stirring. At the same time, HEP-AMA was crosslinked by radical polymerization to form nanogel structure. Finally, nanoparticles were collected by centrifugation (14,000 rpm, 10 min) after 10 h. The collected nanoparticles were washed several times by basic water (pH 8.0, adjusted by NH_3_) and lyophilized to obtain dry nanoparticle powder. The pH sensitive nanoparticles were resuspended into basic water (pH 8.0, adjusted by NH_3_) to form diluted nanoparticle suspension (lower than 100 μg/mL) by ultrasonic probe for 1 min and further characterized by dynamic light scattering (DLS, nano ZS). In morphology investigation, 50 μL of resuspended nanoparticles was dried on silica layer for scanning electron microscope (SEM, S8100), and another 50 μL was dried on copper mesh for transmission electron microscope (TEM, Tecnai 12) at 200 kV.

### *In vitro* Evaluation of VEGF Delivery Property and Bioactivity Performance

VEGF165 was loaded into nanoparticle through absorbance. Briefly, nanoparticle was dispersed in VEGF165 solution. After the mixture was vibrated for 24 h at 4°C to load VEGF165, nanoparticle was centrifuged to remove unloaded growth factor. The loaded amount was calculated by the difference of VEGF165 concentration between before and after growth factor loading, which was determined by ELISA kit. For VEGF165 release assay, 120 mg VEGF165 encapsulated nanoparticle was dispersed in 4 mL PBS under vibration at 37°C. At appropriate intervals, 500 μL released solution was withdrawn after centrifuged and qualified by ELISA kit. Simultaneously, 500 μL fresh solution was supplemented into released solution.VEGF165 concentration of each interval was obtained by referring to the standard curve. The cumulative released VEGF165 was calculated by VEGF165 amount of each interval.

The released VEGF165 was co-cultured with HUVEC cells for its bioactivity evaluation. Briefly, VEGF165 was released in DMEM medium with 30 mg/mL for 12 h. The released medium was collected for further use. HUVEC cells were incubated in a humidified atmosphere of 95% air and 5% CO_2_ at 37°C. The used cells were detached using 0.25% trypsin in PBS for the experiment. Then 100 μL (or 1 mL) of the released medium were added into each well of 96-well (or 12-well) culture plate, into which the 100 μL (or 1 mL) cell suspension with cell density of 2^*^10^4^ cell/mL was subsequently added. Cell viability (MTT assay), DNA content and cell morphology were characterized as a function of cultural time. For MTT assay, 20 μL MTT was supplemented into each well and successively cultured for another 4 h. Then the absorbance of 200 μL MTT/DMSO solution at 560 nm was recorded by a microplate reader (Infinite M200 PRO).For DNA assay, detached cells of each well (12-well culture plate) was digested by 500 μL 10 mg/mL papain solution at 65°C overnight, which was qualified by Quant-iT^TM^ PicoGreen^®^ dsDNA kit. Finally, cells were observed by fluorescence microscope (IX73).

### Hydrogel Formation and Characterization

**Scheme 3:**
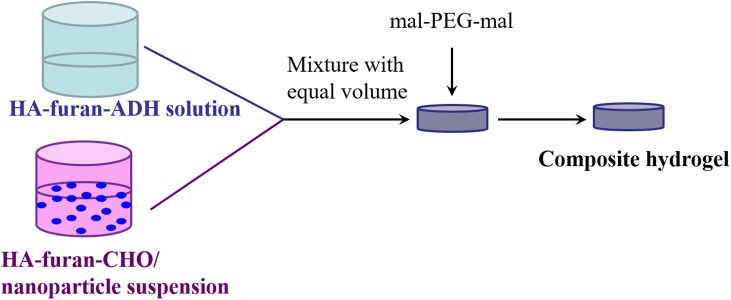
Scheme illustration to show the formation of composite hydrogel.

HA-furan-ADH and HA-furan-CHO were dissolved in water, respectively, to obtain two kind solutions with certain concentration from 2 to 10% w/v. The above-mentioned solutions with same concentration were mixed with equal volume, into which equimolar mal-PEG-mal with furan group on HA derivative was added and stirred. Gelation time was obtained by observation method, which was defined as the interval between mixing and loss of fluidity for mixture. The preparation process for composite nanoparticle was shown in Scheme 3. Briefly, nanoparticle was dispersed in HA-furan-CHO solution with final concentration of 10 mg/mL in advance. Composite hydrogel was then fabricated by the same method for pure hydrogel preparation. Composite hydrogel was characterized by rheological measurement in a parallel platemode using a strain-controlled rheometer (MCR102). The self-healing property was recorded by digital photos. Firstly, the formed hydrogel was cut into two parts completely. Secondly, two separated part was put together with complete interface touch for 24 h. Finally, the self-healed hydrogel was recorded by digital photos.

### *In vitro* Cell Growth in Composite Hydrogel

HUVEC cells was incorporated *in situ* into composite hydrogel during hydrogel formation. Briefly, detached HUVEC cells were added to 10% HA-furan-ADH solution to form cell/precursor suspension with cell density of 2^*^10^6^ cell/mL. The cell/precursor suspension was mixed with another 10% w/v precursor/nanoparticle suspension with 10 mg/mL nanoparticle to form composite hydrogel with cells. Into the above-mentioned solution, mal-PEG-mal was added and mixed. Each cell encapsulated hydrogel carrier with final volume of 200 μL was put in each well of 24-well culture plate and incubated in a humidified atmosphere of 95% air and 5% CO_2_ at 37°C. Cytoviability (MTT assay), DNA content and cell morphology (microscope images) were characterized as a function of cultural time. For MTT assay, 100 μL MTT was supplemented into each well and successively cultured for another 4 h. Then hydrogel was dissolved by 1 mL DMSO, the absorbance of 200 μL above solution at 560 nm was recorded by a microplate reader (Infinite M200 PRO). For DNA assay, each gel with cells was digested by 500 μL 10 mg/mL papain solution at 65°C overnight, which was qualified by Quant-iT™ PicoGreen^®^ dsDNA kit. Finally, cells in hydrogel film were observed by fluorescence microscope (IX73).

### Statistical Analysis

Data were analyzed using the *t*-test for differences. The software of origin was used to calculate these differences for *p* value. Results were reported as means ± standard deviation, at least 3 replicates (from different samples) formed by above-mentioned method were analyzed in all experiments. The sample size for hydrogel is about 200 mg. The significant level was set at *p*<0.05.

## Result and Discussion

### pH-Sensitive Bifunctional Nanoparticle Formation

**Figure 1:**
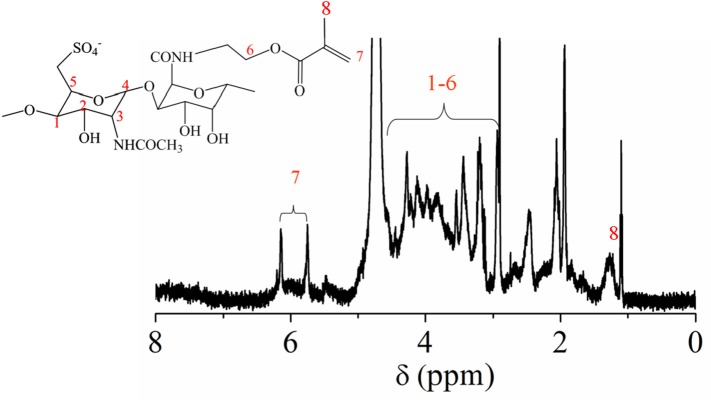
^1^H NMR spectrum of HEP-AMA.

In order to obtain crosslinkable heparin derivative, HEP-AMA was first synthesized and characterized by ^1^H NMR spectrum in Figure 1. The details of chemical shift are listed as follows: the chemical shift at 1.6 ppm is attributed to the protons of methyl group of AMA at 8 position, the chemical shifts from 3.0 to 4.2 ppm are attributed to the protons of pyranose ring and -CH_2_-O from 1 to 6 position, the chemical shifts at 5.6 ppm and 6.2 are attributed to the protons of C = CH_2_ of AMA at 7 position. The chemical shift at 7 position confirmed successful grafting of AMA onto HEP main chain.

**Figure 2:**
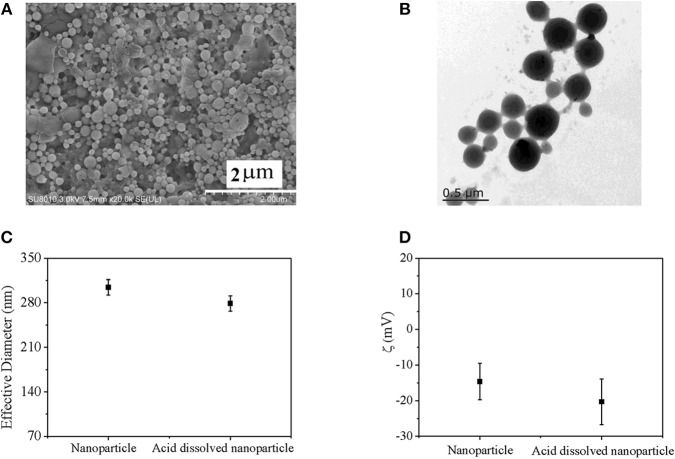
**(A)** SEM image of nanoparticle; **(B)** TEM image of nanoparticle; **(C)** Effective diameter; and **(D)** zeta potential of nanoparticles determined by DLS.

pH-sensitive bifunctional nanoparticle was prepared by double emulsion method combined with *in situ* polymerization (Scheme 2). Final nanoparticle exhibited homogeneous sphere morphology, which was confirmed by SEM image (Figure 2A) and TEM image (Figure 2B). Furthermore, different brightness and obvious boundary was found in the TEM image, which indicated different phase structure. The dark structure might be ascribed to pH sensitive Ac-β-CD, while the light structure might be attributed to HEP nanogel. Simultaneously, slight adhesive structure was also found in the TEM image, which might be due to slight crosslinking of nanogel during the process of *in situ* polymerization and conglutination in drying process of HEP nanogel. These results indicated that nanogel had been successfully synthesized during the preparation of nanoparticle. Moreover, effective diameters and zeta potentials of prepared nanoparticles in PBS and in acid were recorded by DLS method, which was shown in Figures 2C,D. Effective diameter of nanoparticle in PBS was 304 ± 12 nm, which was larger than that in acid solution of 279 ± 12 nm. However, the difference between them had no significant difference. Since pH sensitive property of the bifunctional nanoparticle come from Ac-β-CD dissolution in acid solution due to degradation of Ac-β-CD, which was confirmed by transparency variation of nanoparticle suspension, only HEP nanogel existed in acid solution (Chen et al., 2017, 2018; Wang X. et al., 2018). Since nanogel was formed *in situ* during the fabrication of Ac-β-CD nanoparticle, they had similar diameter, which was also confirmed by the TEM image (Figure 2B). The zeta potential for nanoparticle in acid solution was larger than that in PBS due to contribution of negative heparin in acid environment. However, no significant difference was found between them. The nanoparticle possessed similar chemical component with nanogel so that they had similar zeta potential except slight influence of pH value.

### *In vitro* Evaluation for the Bifunctional Nanoparticle

**Figure 3:**
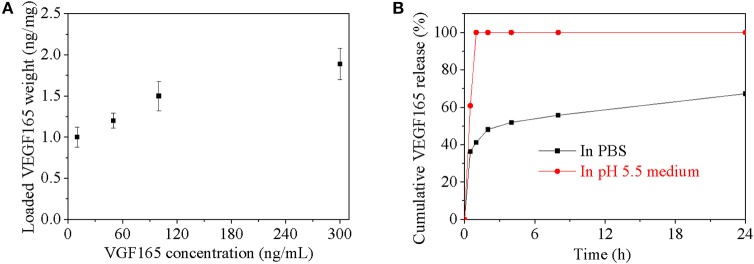
**(A)** Loaded VEGF165 weight in every microgram nanoparticle as a function of VEGF165 concentration; **(B)** Cumulative VEGF165 release in PBS with pH value of 7.4.

The growth factor encapsulated capacity and its release behavior *in vitro* for the bifunctional nanoparticle were investigated in Figures 3A,B. The equilibrium encapsulated growth factor in nanoparticles increased with the initial growth factor's concentration especially when the concentration is lower than 120 ng/mL (Figure 3A). The maximum growth factor encapsulated capacity was about 1.9 ng/mg nanoparticles, which condition was also used for further release behavior investigation. In PBS (pH 7.4), about 65% could be released in 24 h from nanoparticle, about 50% growth factor was burst released in the first 2 h and remaining 15% growth factor was linearly released from nanoparticles in the following 22 h. In mild acid medium (pH 5.5), nearly all the growth factor dispersed homogeneously in the medium after 1 h due to degradation of Ac-β-CD nanoparticle. But it was not sure whether growth factor had been diffused into medium or still incorporated with homogeneous dispersed heparin nanogel.

**Figure 4:**
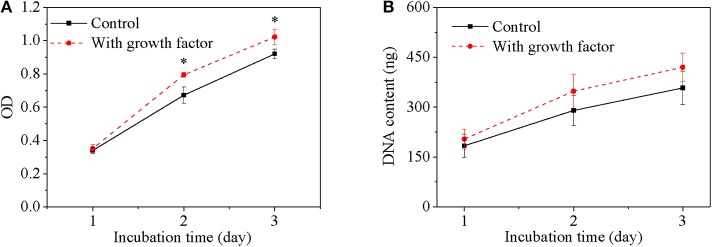
Cell viability **(A)** and DNA content **(B)** of cells as a function of culture time. Cell density is 20,000/mL. Two hundred microliter for MTT assay of 96-well TCPs and 2 mL for DNA assay of 6-well TCPs. *p < 0.05.

**Figure 5:**
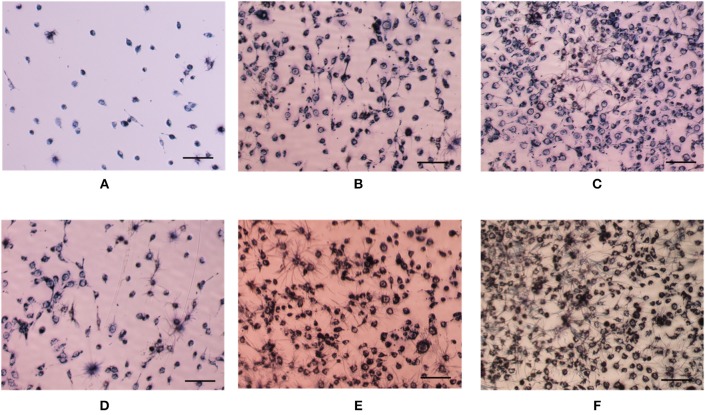
Optical images of HUVEC cells with **(D–F)** or without **(A–C)** growth factor after cultured 1 day **(A,D)**, 2 days **(B,E)**, and 3 days **(C,F)**. Cell seeding density is 4,000/well. Cells were stained by MTT. The scale is 100 μm.

The bioactivity of released growth factor was evaluated by *in vitro* cell behavior using cells without growth factor as a control. Cell viability of the growth factor group increased with culture time just as the control group (Figure 4A). Furthermore, cell viability of the growth factor group was significantly higher than the control group especially after cell had been cultured for 2 days. Since DNA content of each cell is constant, DNA content reflects cell number. DNA content of cells for the two groups increased with culture time, while DNA content of cells for the growth factor group was higher than that for the control group (Figure 4B). However, the increase had no significant difference according to statistical analysis. The space of 2D cell culture for cell growth is limited to a constant value, which may restrict cell proliferation if the space could not accommodate more cells. Moreover, cell morphology was shown in Figure 5. Cell on TCPs exhibited round-like polygon shape regardless culture time and existence of growth factor (Figures 5A–F). It was also found that the number of cells for the growth factor group is larger than that of the control group, which was consistent with results of cell viability and DNA content. Therefore, the released growth factor could promote cell growth from the above-mentioned results, which indicated released growth factor could maintain its bioactivity.

### Fabrication and Characterization of Injectable Composite Hydrogel

**Figure 6:**
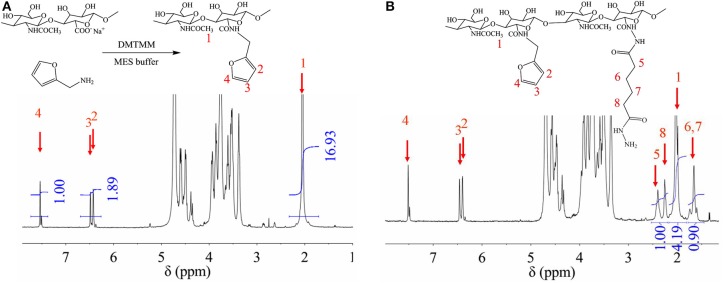
^1^H NMR of HA-furan **(A)**, and HA-furan-ADH **(B)**.

HA-furan and HA-furan-ADH were synthesized and characterized by ^1^H NMR spectrum in Figures 6A,B. Since they are both HA derivatives, chemical shifts from 3.0 to 4.2 ppm in both Figures 6A,B are attributed to the protons of pyranose ring. Other chemical shifts for HA-furan (Figure 6A) are listed as follows: the chemical shift at 1.9 ppm is attributed to the protons of CH_3_-O at 1 position, and the chemical shifts at 6.4, 6.5, and 7.5 ppm are attributed to the protons of furan ring at 2–4 position. The chemical shift at 2–4 position confirmed successful grafting of furan onto HA main chain. Since the H number of 2–4 position in each molecule and CH_3_-O group every two pyranose rings are fixed, their relative area ratio could be used to calculate the substitute degree of furan group on HA, which was 17%. Besides Figure 6A mentioned, chemical shifts at 1.7, 2.2, and 2.4 ppm for HA-furan-ADH (Figure 6B) are attributed to -CH_2_ groups at 5–8 position, which confirmed successful grafting of ADH onto HA-furan chain. Similarly, the substitute degree of ADH was calculated to be 17%. In addition, HA-furan-CHO was confirmed and quantified by t-butyl carbazate assay. The substitute degree of CHO was calculated to be 21%.

**Figure 7:**
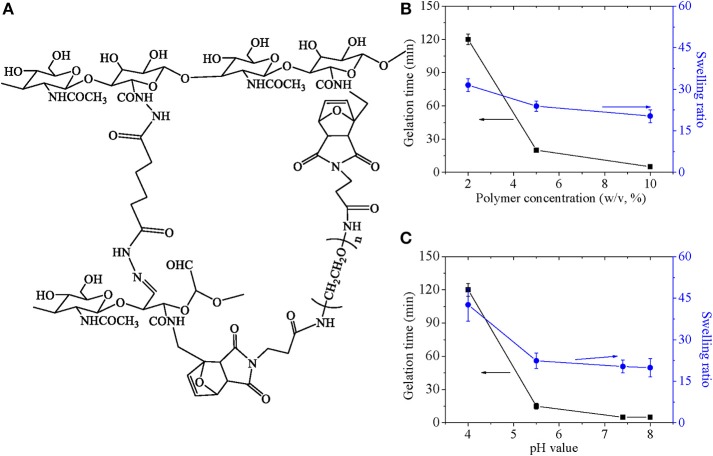
**(A)** The mechanisms for the formation of the hydrazine bond and for the Diels-Adler reaction. Gelation time and swelling ratio as a function of **(B)** polymer concentration and **(C)** pH value.

In the hydrogel system, two kinds of crosslinking points were formed to strengthen polymer network, as shown in Figure 7A. One was acyl hydrazone bond formed by CHO and ADH, which was a dynamic covalent and sensitive to pH value. The other was formed by reversible diels-alder click chemistry between furan groups and maleimide groups. Also, properties of hydrogel were related with the double crosslinked network, which was discussed as follows. Gelation time and swelling ratio as a function of polymer concentration as well as pH value were investigated, which was shown in Figures 7B,C. Gelation time decreased rapidly along with polymer concentration, and swelling ratio decreased slightly with increase of polymer concentration (Figure 7B). Just as our previous research discussed, enlarged crosslinking point in definite volume increased percentage of reaction between functional groups and accelerate reaction speed, which influenced gelation time and swelling ratio. Gelation time and swelling ratio decreased significantly with decrease of pH value until pH5.5 (Figure 7C). Since acyl hydrazone bond was formed at high pH value medium and broken at low pH value medium with critical point of pH 4.0, it was easily understood that gelation time prolonged due to lack of one kind of crosslinkings in low pH value and swelling ratio enlarged due to less crosslink points. In summary, adjustable gelation time ensured the injectable property for composite hydrogel.

**Figure 8:**
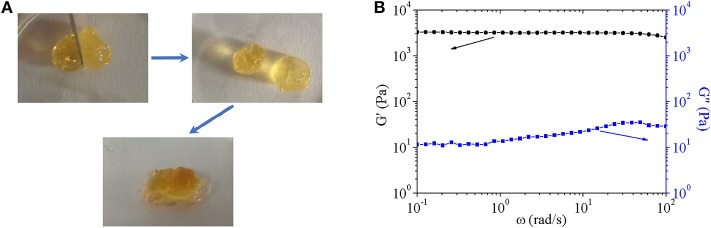
**(A)** Digital images to show self-healing properties of hydrogel. The process included (1) cutting the formed hydrogel; (2) separating two part of above-mentioned hydrogel completely; (3) putting together of two separated part with complete interface touch for 24 h. **(B)** Storage modulus and loss modulus of composite hydrogel.

Furthermore, the self-healing property was confirmed by Figure 8A. Just as above discussed, the hydrogel was crosslinked by dynamic covalent and reversible Diels-alder click chemistry, which endowed hydrogel flexible and reversible crosslinking points. The characteristic gave self-healing property to hydrogel.

Finally, composite hydrogel was characterized by gelation time and swelling ratio, which possessed similar properties to pure hydrogel. Additionally, viscoelastic behaviors of composite hydrogels were shown in Figure 8B. Storage moduli of hydrogel was around 3 ×10^3^ Pa, which was 100–300 times higher than loss moduli of 100–300 Pa over the frequency range of 10^−1^-10^2^ rad/s. The result indicated that composite hydrogels had typical characteristics of elastomers. With increasing angular frequency, storage moduli showed little decrease and loss moduli increased gently with no sign of breakage as far as the measured angular frequency range was concerned. The mechanical property ensured its potential application as a scaffold.

Moreover, the degradation of both pure hydrogel and composite hydrogel was about 3 weeks. No significant difference for degradation time was found between hydrogel and composite hydrogel.

### *In vitro* Evaluation for Composite Hydrogel

**Figure 9:**
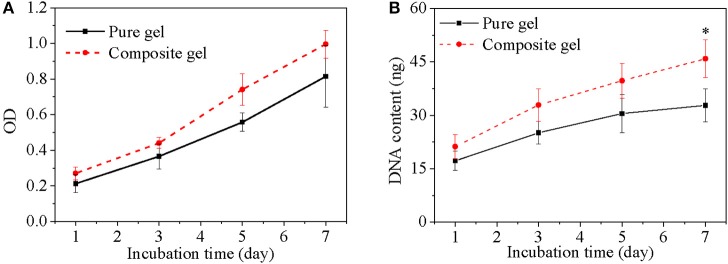
Cell viability **(A)** and DNA content **(B)** of cells in different gels as a function of culture time. cell density is 400,000/gel. *p < 0.05.

**Figure 10:**
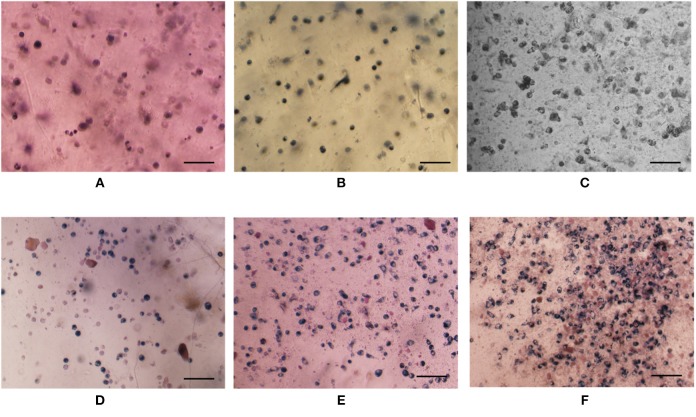
Optical images of cells in gels without **(A–C)** or with **(D–F)** growth factor encapsulated nanoparticle after cultured 1 day **(A,D)**, 3 days **(B,E)**, and 5 days **(C,F)**. Cell seeding density is 400,000/gel. Cells were stained by MTT. The scale is 100 μm.

Cell was incorporated into composite hydrogel to evaluate its biomedical application using pure hydrogel without nanoparticle as a control. Cell viability both in either pure hydrogel and in composite hydrogel increased obviously along with culture time and the increase have statistically significant difference (Figure 9A), which indicated that either pure hydrogel or composite hydrogel can support cell growth. Moreover, cell viability in composite hydrogel was higher than that in pure hydrogel especially after cells were cultured for 3 days (Figure 9A). Especially, for 5 and 7 days, the difference between composite hydrogel and pure hydrogel has obvious statistical significance. Higher cell viability in composite hydrogel was ascribed to encapsulated growth factor. Similarly, DNA content of either pure hydrogel or composite hydrogel increased significantly along with culture time. While DNA content of composite hydrogel was higher than that of pure hydrogel especially after cells were cultured for 3 days (Figure 9B). Just as above discussed, DNA content reflects cell number. Hence hydrogels could support cell proliferation and composite hydrogel could accelerate cell proliferation more. Furthermore, cells in hydrogels exhibited round morphology, which was recorded by microscope in Figure 10. At day 1, some round cells homogenous dispersed in hydrogels (Figures 10A,D); at day 3, homogenous dispersed cells increased (Figures 10B,E); at day 5, cells increased further (Figures 10C,F). Overally, cell number in composite hydrogel (Figures 10D–F) was obvious larger than that in pure hydrogel (Figures 10A–C). Therefore, these results were consistent and confirmed that hydrogels could maintain cell activity and support cell growth, and composite hydrogel possessed better properties.

## Conclusion

pH-sensitive bifunctional nanoparticle was successfully prepared by combined W/O/W technique and *in situ* polymerization. Final nanoparticle exhibited homogeneous sphere morphology and biphase structure. pH sensitive property of the nanoparticle was confirmed by effective diameter change from 314 nm in PBS to 299 nm in acid solution as well as zeta potential change. The growth factor encapsulated capacity in the nanoparticle was influenced by initial growth factor concentration with maximum encapsulated amount of 1.9 ng/mg nanoparticle. Their release behaviors were dependent on pH value of released medium. Detailedly, about 60% could be released in 24 h in PBS; but nearly all the growth factor dispersed homogeneously in pH 5.5 medium after 1 h. *In vitro* investigation including cell viability, DNA content and cell morphology revealed that the released growth factor could increase cell viability and promote cell growth. In further step, HA-furan, HA-furan-ADH, and HA-furan-CHO were successfully synthesized with furan substitute degree of 17%, ADH substitute degree of 17%, CHO substitute degree of 21%. Hydrogel was crosslinked by dynamic covalent and reversible diels-alder click chemistry, which endowed hydrogel flexible and adjustable properties including self-healing property. Gelation time and swelling ratio were influenced by pH value and polymer concentration. Higher polymer concentration or higher pH value resulted in shorter gelation time and smaller swelling ratio. After nanoparticle was incorporated into hydrogel, composite hydrogel exhibited acceptable mechanical property as a scaffold for biomedical field with storage moduli of 3 ×10^3^ Pa and loss moduli of 100–300 Pa. *In vitro* evaluation from viability, DNA content and cell morphology results confirmed that hydrogels could maintain cell activity and support cell growth, and further composite hydrogel possessed better properties.

## Data Availability

The raw data supporting the conclusions of this manuscript will be made available by the authors, without undue reservation, to any qualified researcher.

## Author Contributions

XH provided an idea, designed the whole research, and write the manuscript. ZG synthesized and characterized materials, give evaluations for the injectable composite hydrogel. HT helped to perform *in vitro* evaluation. HW prepared nanoparticle and helped to culture cells. XM helped to synthesize and characterize materials. JP helped to characterize materials.

### Conflict of Interest Statement

The authors declare that the research was conducted in the absence of any commercial or financial relationships that could be construed as a potential conflict of interest.
